# Depressive symptoms and oral mucositis in children with oncological diseases: a cross-sectional study

**DOI:** 10.1590/1807-3107bor-2024.vol38.0033

**Published:** 2024-05-13

**Authors:** Felipe Barreto LEMOS, Andressa Chang Fernandes Rodrigues da SILVA, Fernanda Pereira LIMA, Fernanda Conceição MACHADO, Luanderson Lopes PEREIRA, Arnaldo França CALDAS, Bruna Bustani dos SANTOS, Andréia Cristina Leal FIGUEIREDO

**Affiliations:** (a) Universidade Federal da Bahia – UFBA, School of Dentistry, Departament of Social Dentistry and Pediatrics, Salvador, BA, Brasil.; (b) Universidade Federal de Pernambuco – UFPB, School of Dentistry, Department of Clinical and Preventive Dentistry, Recife, Pernambuco, Brasil.; (c) Martagão Gesteira Hospital. Álvaro Bahia League against Child Mortality, Salvador, BA, Brasil.

**Keywords:** Neoplasms, Stomatitis, Depression

## Abstract

The aim of this study was to investigate the correlation between depressive symptoms and the occurrence of oral mucositis in children with oncological diseases treated at a reference hospital. This was a cross-sectional study conducted with individuals aged 4 to 18 years, diagnosed with primary neoplasms. Data was collected by using a questionnaire that assessed the degree of oral mucositis according to the World Health Organization index, the risk of oral mucositis according to the Child’s International Mucositis Evaluation Scale, and depressive symptoms using the Children’s Depression Inventory. The data were analyzed and subjected to Spearman’s correlation, chi-square test, and Fisher’s exact test, considering p<0.05. A statistically significant correlation was observed between depressive symptoms and the degree of oral mucositis (p = 0.044), and also between the “pain” variable within the risk of oral mucositis and depressive symptoms (p = 0.021). Based on the findings, it can be inferred that oral mucositis may be associated with the development of depressive symptoms and may be influenced by the individual’s hospitalization, thereby affecting the quality of life of pediatric patients.

## Introduction

Although rare, childhood cancer encompasses a heterogeneous group of neoplasms that are still underreported, especially in underdeveloped countries. Moreover, they are an important cause of mortality and morbidity among children and adolescents.^1^

In this context, there has been a noticeable increase in hospitalized pediatric patients experiencing moderate to severe depression. This condition often arises due to the side effects of cancer therapy, which include sleep disturbances, hair loss, nausea, fever, pain, and oral mucositis, among other prevalent oral manifestations.^2,3^ It may also be caused by abnormal activation of the inflammatory system and its subsequent impact on the regulation of neurotransmitter production. This has been attributed to the action of cytokines such as IL-1β, IL-6, and TNF-α, observed in pediatric patients with major depressive disorder.^3,4^

Among oncology patients, depressive symptoms are a common reality, ranging from 8% to 24% across various types of cancers and treatment phases^5^ This association between cancer and depression stems from factors that begin at the time of cancer diagnosis and intensify during treatment, owing to repeated hospitalizations, side effects, disrupted plans, and decreased quality of life.^4^

Although oncological treatment in children and adolescents has been linked to the presence of more intense oral mucositis, with symptomatic implications directly affecting their quality of life, the contemporary literature lacks robust studies investigating potential associations and risk factors between the onset of oral mucositis lesions and the development of depressive symptoms. The aim of this study was to assess the relationship between depressive symptoms and the occurrence of oral mucositis in pediatric patients undergoing oncological treatment.

## Methodology

### Study design

The study consisted of observational cross-sectional research conducted with both pediatric oncology inpatients and outpatients at a reference hospital located in Bahia, Brazil. The study was designed in accordance with the STROBE (Strengthening the Reporting of Observational Studies in Epidemiology) checklist and was approved by the Research Ethics Committee of the School of Dentistry at the Federal University of Bahia – Approval No. 4,834,633. All participants and their legal guardians provided signed Terms of both Informed Consent and Assent.

### Sample size calculation

The sample size was calculated using Epi Info software, version 7.1.0 (CDC, Atlanta, USA, 2012). The parameters considered were 80% power, a frequency of 60% for the exposed group, an odds ratio of 1.5, and a confidence level of 95%. The sample size calculated was 103 participants.

### Inclusion and exclusion criteria

Inclusion criteria involved individuals aged 4 to 18 years, hospitalized or receiving outpatient care at the hospital, with a medical diagnosis of primary malignant neoplasia. These patients had undergone treatment involving surgery, chemotherapy, or radiotherapy. Exclusion criteria stipulated individuals with concurrent systemic diseases alongside malignant neoplasia, those exhibiting alterations in the oral cavity such as autoimmune diseases or a diagnosis of acquired immunodeficiency syndrome (AIDS), and participants with severe intellectual disabilities.

### Scale for oral mucositis

Participants who met the inclusion criteria were evaluated over a period of six months. Oral cavity inspections consisted of the assessment of oral structures, including upper and lower lips, tongue, floor of the mouth, cheeks, hard and soft palates, and gums.

Two scales were used to determine the degree of oral mucositis. The Child’s International Mucositis Evaluation Scale (ChIMES) that includes seven items to measure topics such as pain, swallowing of solid and liquid foods and saliva, use of pain medications, and perception of oral lesions. Scores ranged from 0 to 5 for the first four questions, with 0 representing the absence of symptoms and 5 representing the worst symptoms. The last three questions were scored as 0 or 1 based on negative or positive statements regarding medication use and lesion appearance. The final score was calculated by dividing the score obtained by the maximum possible score of 23 and then multiplying it by 100^6^.

The second scale used was the World Health Organization’s (WHO) Oral Toxicity Scale, which assigns scores of 0-4 to signs and symptoms for determining the grade of oral mucositis. Based on this score, grades were determined as follows: 0 for no signs and symptoms, 1-2 for mild to moderate mucositis, and 3-4 for severe mucositis. Participants identified with mucositis lesions were referred to the dental team for treatment with laser therapy.

### Scale for depressive symptoms

Furthermore, the depressive symptoms of participants were assessed by using the Childhood Depression Inventory (CDI). This self-assessment scale, widely used in non-clinical populations, provides insights into depressive behaviors in a simple and time-efficient manner. Scores ranged from 0 to 40 based on statements expressed in 20 phrases adapted to the Brazilian context. A cutoff score of 16 points was established for determining the significance of depressive symptoms^7^. For participants aged 4 to 8, caregivers responded to the modified questionnaire on their behalf, while those older than 8 years answered individually.

The researchers identified participants who presented depressive symptoms by means of the scales, transmitted the pertinent information to the hospital psychologists, and these patients were monitored by the hospital’s multidisciplinary service that included psychologists, psychiatrists, and occupational therapists.

### Data analysis

All data collected were tabulated using SPSS software version 25.0, subjected to descriptive statistical analysis, and measures of central tendency and dispersion were calculated. Certain variables were dichotomized to improve statistical analysis. The normality of distribution for variables was tested using the Kolmogorov–Smirnov test. To correlate oral mucositis and depression, the Spearman test was applied, using the ordinal scales WHO, CHIMES, and CDI, while qualitative variables were compared using the chi-square and Fisher’s exact tests, all considering a significance level of 95% (p < 0.05).

## Results

A total of 105 children and adolescents were selected to participate in the study based on the inclusion and exclusion criteria. The mean age of these children and adolescents was 9.7 years (Standard Deviation = 4.2). In terms of gender, there were 53 (50.5%) male individuals. Out of the 105 participants interviewed, only seven (6.7%) children with oral mucositis were identified, according to the WHO scale. However, 40% (n = 42) showed some risk of developing oral mucositis based on the criteria established by the ChIMES scale.

Relative to the ChIMES scale, a statistically significant difference (p < 0.05) was observed between the treatment setting and indication of risk for the development of mucositis. Among those undergoing outpatient treatment, 19.5% were at risk of developing mucositis, compared with 53.1% of hospitalized patients. No statistically significant associations were found between hematological toxicity and the treatment used, including the use of methotrexate (p > 0.05) (Table).

When calculating the correlation between depressive symptoms and oral mucositis, a statistically significant result was observed between the data from the CDI index and the ChIMES scale. However, the same significance was not observed when data were correlated with the WHO scale, as shown in Figures A and B.

Correlations were also calculated between two subgroups of the ChIMES index – pain and function. A positive correlation was observed between the “pain” subgroup and depressive symptoms (p < 0.05). However, the same pattern was not found when calculating the correlation with the “function” subgroup (p > 0.05), as shown in Figures C and D.

## Discussion

The impact of chronic illness on the daily lives of children and adolescents have been evidenced by symptoms that can affect treatment of the disease. However, mental health conditions are often not well-documented, and little is known about the frequency and persistence of depressive symptoms in this population.^8^

The main causes attributed to the development of these symptoms have been rooted in social and familial distancing, as well as the direct consequences of adverse effects on patients undergoing cancer treatment, such as hair loss, nausea, and pain.^9^ Pain and depressive symptoms can coexist, and studies have indicated evidence confirming this correlation, showing exacerbation or recurrence of depressive symptoms in patients with chronic pain.^10,11^ These effects suggested a higher likelihood of recurrence and worsened prognosis of neoplasms in younger patients.^9,11^

Children and adolescents may experience more anxiety and depression symptoms during chemotherapy sessions than after treatment.^8,12^ Around 49% to 62% of children experience constant pain during antineoplastic treatment, which can pose a significant risk to their mental health.^13^ The painful symptomatology of oral mucositis has shown evidence of having direct impact on the quality of life of children and adolescents since it affects their ability to chew, swallow, articulate, and cause pain.^2^

In addition to these factors, hospitalization of the patients investigated in this study also appeared to have an influence the onset of oral mucositis. Hospitalization of children and adolescents with cancer also appears to play an important role in the development of depressive symptoms. The increased prevalence of physical and psychosocial suffering, including pain, nausea, fatigue, and changes in appetite are common symptoms experienced during hospitalization for chemotherapy. A study with hospitalized children with cancer emphasized that the hospital environment significantly disrupted the individuals’ sleep, resulting in nighttime awakenings and insomnia, which further contributed to fatigue.^14^

The combination of these factors represents a significant challenge to individuals with oncological diseases, their families and to the multidisciplinary team, due to the complexity of diagnosis and management.^10^ Children and adolescents who exhibit depression associated with painful symptoms often tend to isolate themselves socially and experience difficulties in communicating with their loved ones.^14^

These conditions require specific monitoring by the entire multidisciplinary team to prevent deterioration of the clinical conditions and to maintain nutritional support.^15^ As integral members of the oncology multidisciplinary team, dentists should follow up patients right from the time of cancer diagnosis through to the post-treatment period, according to their individual needs. The impact of oral cavity involvement on oncological treatment can be significant, with both acute and chronic aspects affecting the individual across various dimensions.^16^

Although the results of this study indicated an association between pain, oral mucositis, and depressive symptoms in pediatric patients, further longitudinal studies that also investigate the duration of these patients’ treatment are necessary for a more comprehensive understanding of this hypothesis.

## Conclusion

Based on the findings, a correlation was observed between depressive symptoms and the degree of oral mucositis reported in the sample. Furthermore, a correlation between pain symptomatology and the risk of these lesions was identified. Moreover, within the sample, a potential association with the influence of hospitalization during oncological treatment was noted.

**Figure 1:**
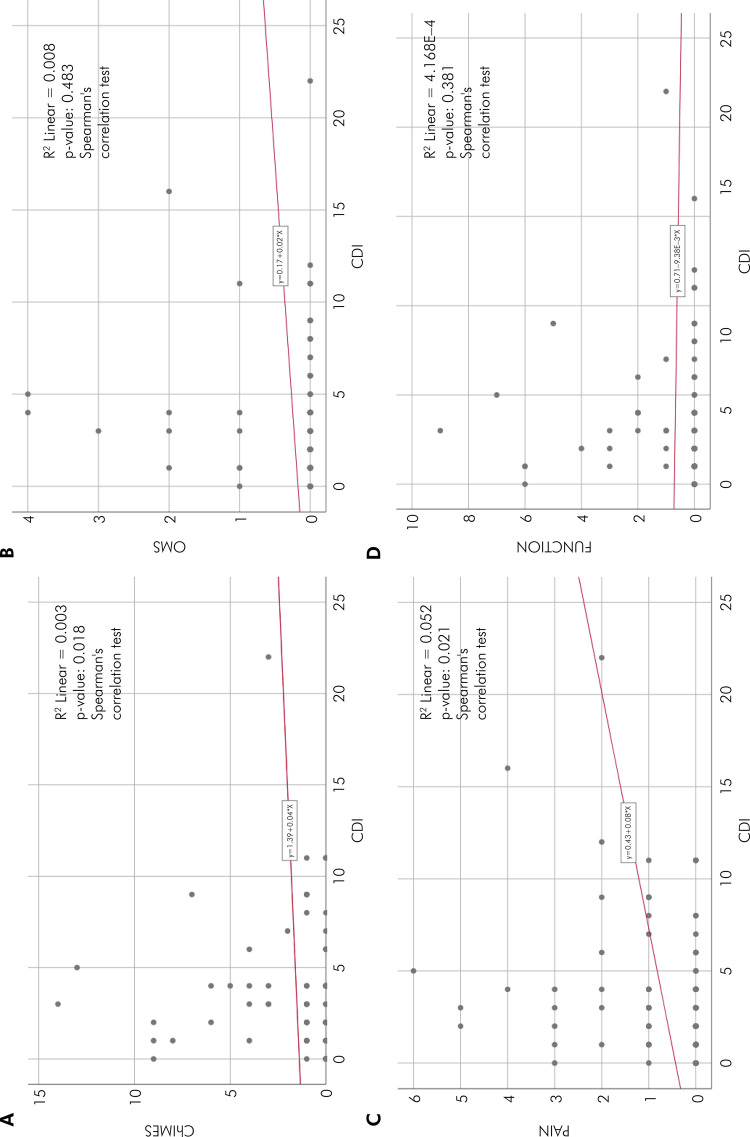
Correlations between variables. A. Correlation between depressive symptoms (CDI) and risk of oral mucositis (ChIMES); B. Correlation between depressive symptoms (CDI) and degree of oral mucositis (WHO scale); C. Correlation between depressive symptoms (CDI) and the subcategory “PAIN” (ChIMES); D. Correlation between depressive symptoms (CDI) and the subcategory “FUNCTION” (ChIMES).

**Table 1:** Correlation between factors linked to oncological disease and the presence of oral mucositis in the sample.

Variable	WHO				Chimes			

	n (%)	n (%)	p-value	OR [95%CI]	n (%)	n (%)	p-value	OR [95%CI]
	
	Absence 98 (93,3)	Presence 7 (6,7)			No risk n 63 (60)	Risk 42 (40)		
Treatment location								
Hospitalized 64 (61)	58 (90,6)	06 (9,4)	0,162**	0,242 [0,028–2,085]	30 (46,9)	34 (53,1)	0,001*	0,214 [0,086–0,534]
Outpatient ward 41 (39)	40 (97,6)	01 (2,4)			33 (80,5)	08 (19,5)		
Use of Methotrexate								
No 96 (91,4)	59 (92,7)	07 (7,3)	...	...	58 (60,4)	38 (39,6)	0,520**	1,221 [0,308–4,839]
Yes 9 (8,6)	09 (100)	0 (0)			05 (55,6)	04 (44,4)		
Hemotological toxicity								
Normal 82 (78,1)	78 (95,1)	04 (4,9)	0,176**	2,925 [0,605 –14,137]	50 (61)	32 (39)	0,700*	1,202 [0,471–3,065]
Neutropenic 23 (21,9)	20 (87)	02 (13)			13 (56,5)	10 (43,5)		
Type of treatment								
Radiotherapy 7 (6,7)	07 (100)	0 (0)	...	...	04 (57,1)	03 (42,9)	0,584**	0,881 [0,187–4,155]
Chemotherapy 94 (93,3)	91 (92,9)	07 (7,1)			59 (60,2)	39 (39,8)		

*Chi-square test; **Fisher’s exact test.
